# Changing of the Guard

**DOI:** 10.3402/ehtj.v6i0.20449

**Published:** 2013-02-08

**Authors:** Nathaniel Hupert

In the five years since the *Emerging Health Threats Journal* published its first issue, a steady stream “100-year” events has increased awareness of the multitude of threats to human health. This month, the Journal is announcing a change at the helm: I will be taking over the position of Chief Editor from Andrew Robertson, who was instrumental in crafting the *Journal's* unique vision and provided exemplary leadership from its founding. Dr. Robertson has graciously agreed to transition to Associate Editor, in which role he will continue to help chart the *Journal's* course through the increasingly turbulent waters of world health crises.

In his first editorial, Dr. Robertson made the case for the *Journal*'s focus on health threats *generally*, noting that they encompass “new and evolving health issues that society faces on a daily basis, including new infectious diseases, chemical and environmental hazards, radiation exposure, disasters, and cultural and population health issues.” A new journal, he wrote, was needed “to provide comprehensive reviews and original papers for a wide audience, particularly in an open-access format.” This focus on predicting, mitigating, and responding to newly pressing threats to human health remains at the core of the *Journal*'s mission, while a commitment to open-access publication remains the cornerstone of our production philosophy.

As is likely the case with much of our readership, over the last decade I have experienced a number of emerging health threats firsthand both as a citizen and as a health professional living in New York City. These events have ranged from large-scale terrorist bombings, bioterrorism, and environmental contamination in 2001–2002, to pandemic influenza in 2009–2010, to current hurricane-related disruptions of major hospital systems that continue to jeopardize health care provision in the city months after the storm. As you read this, those of you living in Asia, Africa, South America, Oceania, and Europe, whether in urban or rural settings, likely are thinking of similar lists of health crises large and small that have befallen your communities over this period. My hope is that this journal will become an increasingly vital part of the international conversation about how to predict and prevent these threats, as well as how to better mitigate and recover from them when they do occur.

An increased emphasis on this latter point—response to and recovery from health crises—is one change in editorial emphasis that I hope to bring to the *Journal*. There are a number of scientific fields, ranging from risk sciences to operations research and information engineering, that are critical to successful response to novel and well-known threats but that are not yet well integrated into public health or health care emergency response (see Figure). Bridging the divides among these fields will require collaborative exploration of threat spaces and also collaborative work in actual response operations. Because of its unique focus and format, I believe the *Emerging Health Threat Journal* can become an important forum for this effort on the international stage, and I welcome contributions from those of you who share this vision.

**Fig. 1:**
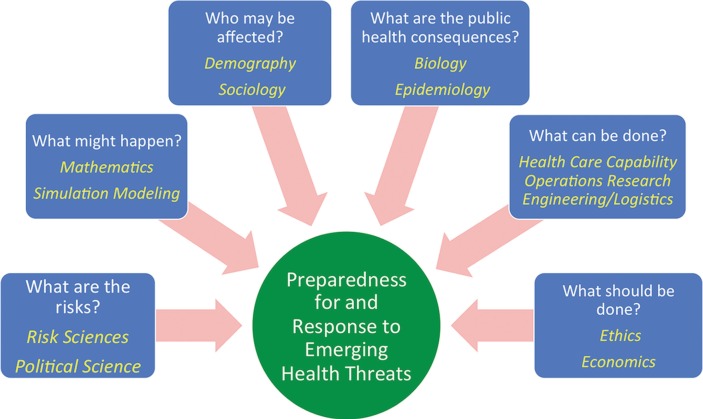
The intellectual landscape of emerging health threats. (Adapted from Hupert, N. Predicting and planning for public health disasters. In: Levy B, Sidel V, editors. Terrorism and public health. New York: Oxford University Press; 2011.)

Another area of increased focus will be the varied (and incompletely understood) effects of the global environment on the emergence of and response to health hazards. As noted by Redlener and Reilly in their commentary after Hurricane Sandy, “climate change, with resultant changes in sea level and weather patterns, will make more frequent and severe storms a grim reality in the years ahead.”^1^ Mapping out the impact of these changes on "routine" hazards, as well as delineating their role in truly emergent threats, will be increasingly important for the scientific and policy communities.

Try as we might to make it otherwise, health threats and their resulting crises are an integral part of the fabric of human life. In many traditions across the globe, catastrophe is considered both a calamity and an opportunity. One of the great Hindu deities, Shiva, is known as “the Destroyer” but also “the Transformer;” only such a liminal deity could provide both an epitaph for the Manhattan Project (“Now I am become Death, the destroyer of worlds”) and also a fitting subject for a statue outside of the CERN European Laboratory for Particle Physics. Our challenge is to draw knowledge, lessons, and even strength from the calamities that have befallen us and will yet do so, to make our communities healthier, safer, and more conducive to the flourishing of society in harmony with our natural environment. It is a lofty goal, and we may have only a small part to play, but it is a worthy endeavor, and I look forward to the journey with you.

*Nathaniel Hupert, MD, MPH* Chief Editor

## Footnotes

1 Redlener I, Reilly MJ. Lessons from Sandy—Preparing Health Systems for Future Disasters. N Engl J Med 2012; 367: 2269–2271. DOI: 10.1056/NEJMp1213486.

